# Finished sequence and assembly of the DUF1220-rich 1q21 region using a haploid human genome

**DOI:** 10.1186/1471-2164-15-387

**Published:** 2014-05-20

**Authors:** Majesta O’Bleness, Veronica B Searles, C Michael Dickens, David Astling, Derek Albracht, Angel C Y Mak, Yvonne Y Y Lai, Chin Lin, Catherine Chu, Tina Graves, Pui-Yan Kwok, Richard K Wilson, James M Sikela

**Affiliations:** Department of Biochemistry and Molecular Genetics, Human Medical Genetics and Neuroscience Programs, University of Colorado School of Medicine, 12801 E. 17th Avenue, Aurora, CO 80045 USA; The Genome Institute at Washington University School of Medicine, St. Louis, MO 63108 USA; Institute for Human Genetics, University of California San Francisco, San Francisco, CA 94158 USA

**Keywords:** 1q21, DUF1220 domain, Hydatidiform mole

## Abstract

**Background:**

Although the reference human genome sequence was declared finished in 2003, some regions of the genome remain incomplete due to their complex architecture. One such region, 1q21.1-q21.2, is of increasing interest due to its relevance to human disease and evolution. Elucidation of the exact variants behind these associations has been hampered by the repetitive nature of the region and its incomplete assembly. This region also contains 238 of the 270 human DUF1220 protein domains, which are implicated in human brain evolution and neurodevelopment. Additionally, examinations of this protein domain have been challenging due to the incomplete 1q21 build. To address these problems, a single-haplotype hydatidiform mole BAC library (CHORI-17) was used to produce the first complete sequence of the 1q21.1-q21.2 region.

**Results:**

We found and addressed several inaccuracies in the GRCh37sequence of the 1q21 region on large and small scales, including genomic rearrangements and inversions, and incorrect gene copy number estimates and assemblies. The DUF1220-encoding *NBPF* genes required the most corrections, with 3 genes removed, 2 genes reassigned to the 1p11.2 region, 8 genes requiring assembly corrections for DUF1220 domains (~91 DUF1220 domains were misassigned), and multiple instances of nucleotide changes that reassigned the domain to a different DUF1220 subtype. These corrections resulted in an overall increase in DUF1220 copy number, yielding a haploid total of 289 copies. Approximately 20 of these new DUF1220 copies were the result of a segmental duplication from 1q21.2 to 1p11.2 that included two *NBPF* genes. Interestingly, this duplication may have been the catalyst for the evolutionarily important human lineage-specific chromosome 1 pericentric inversion.

**Conclusions:**

Through the hydatidiform mole genome sequencing effort, the 1q21.1-q21.2 region is complete and misassemblies involving inter- and intra-region duplications have been resolved. The availability of this single haploid sequence path will aid in the investigation of many genetic diseases linked to 1q21, including several associated with DUF1220 copy number variations. Finally, the corrected sequence identified a recent segmental duplication that added 20 additional DUF1220 copies to the human genome, and may have facilitated the chromosome 1 pericentric inversion that is among the most notable human-specific genomic landmarks.

## Background

A major landmark in the modern era of medical genomics research is the sequence and assembly of the human genome. The current genome build, however, contains numerous gaps and areas of potential misassembly. Completion of an accurate assembly is a continuing challenge given the presence of multiple highly duplicated and complex regions that remain largely intractable to analysis with commonly used assembly techniques [1]. Nonetheless, finishing these regions has significant implications for identifying causative disease loci and in turn efficacious treatments for patients with genetic and genomic disorders [2]. The 1q21 region of chromosome 1 is a classic example, given its association with multiple clinical disorders and its complex architecture, with multiple regions of duplication that make complete assembly extremely difficult. The 2009 human genome assembly reflects this challenge, containing 14 gaps in the 7.7 Mb 1q21.1-2 region.

Closing these gaps in the current 1q21 build is a particularly pressing problem given that recurrent genetic and genomic variations in this region have been implicated in a multitude of disease phenotypes: neuropsychiatric diseases such as autism [3, 4] and schizophrenia [5, 6], microcephaly and macrocephaly [7, 8], cardiac conduction and structural defects [9, 10], multiple congenital anomalies [11–13], and ocular deficits [8]. Additionally, this region contains multiple Neuroblastoma Breakpoint Family (*NBPF)* genes encoding 238 of the known 270 copies of DUF1220, a protein domain that has undergone a striking copy number increase specifically in the human lineage [14, 15]. While this extreme DUF1220 copy number increase has been linked to the evolutionary expansion of the human brain [16, 17], the many interspersed and tandem DUF1220 paralogs in the 1q21 region are thought to be major contributors to 1q21 genomic instability leading to numerous disorders [16]. Ascertaining the exact involvement of DUF1220 and other 1q21 sequences in these diseases has been hindered by the incomplete nature of the 1q21 assembly and of the DUF1220-encoding gene family (*NBPF*) in particular. Without a complete, accurate assembly, genotype-phenotype associations are difficult to identify, and those that are found may not provide a complete picture of disease etiology and in some cases may even be incorrect and misleading.

To pursue the completion of the 1q21 genomic region, a haploid hydatidiform mole (CHM1) genome was utilized which reduces the challenges introduced by using a diploid, polymorphic genome [18]. Using bacterial artificial chromosomes (BACs) produced from the CHM1 genome the 14 gaps that remained in this region were closed and a single haploid genomic path generated that spans the 1q21.1-2 region. This new, completed assembly was used to more precisely analyze genomic structural variation in individuals with 1q21 CNVs and microcephaly or macrocephaly.

## Results and discussion

### Sequence finishing and assembly

**Figure 1:**
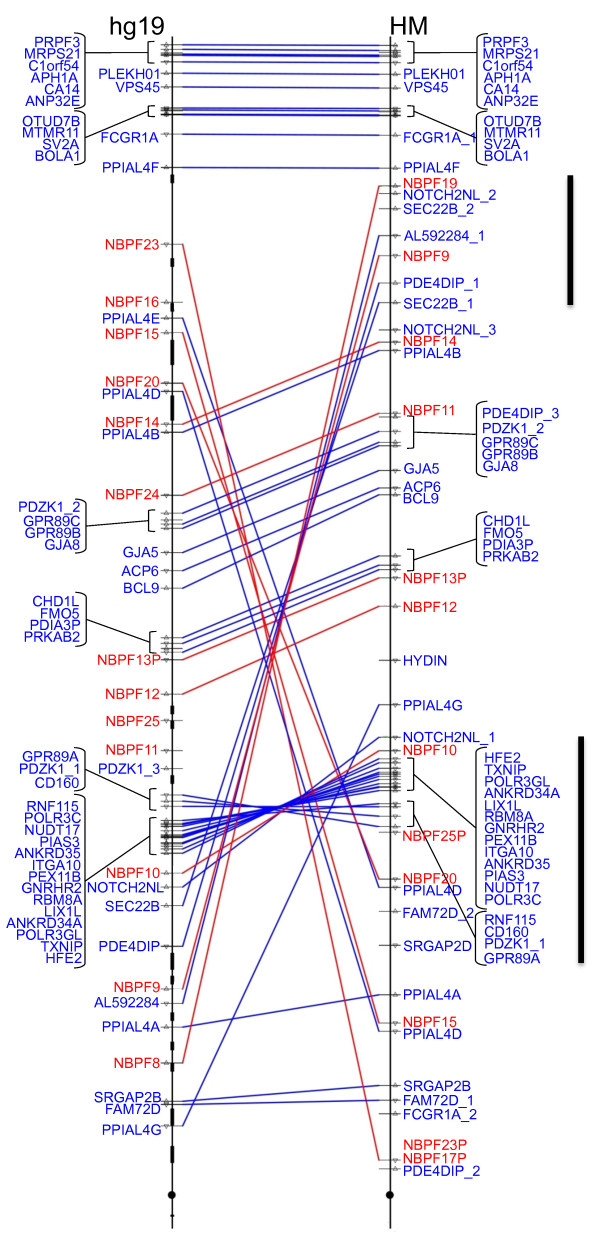
**Comparison of GRCh37/hg19 assembly (left) with the WUSTL CHM1 assembly (right).** *NBPF* genes are indicated in red, all other genes are in blue. Black boxes on the GRCh37 map denote gaps. The vertical bar to the right of the CHM1 map denotes the novel inversion spanning multiple genes discussed in the text.

A total of 48 BACs were sequenced generating a contig of 7,283,150 bp, covering the 1q21.1-q21.2 region of interest. This successfully closed all 14 gaps in the GRCh37/hg19 assembly, added 616,581 bp of sequence, and resulted in the addition of 12 new genes. In addition to these sequence additions, numerous differences between the previous 1q21 assembly and the CHM1 assembly were discovered, including changes in gene order, 2 inverted loci spanning multiple genes, and gene loss associated with multicopy genes (Figure 1, Table 1). While confirmation testing conducted on the assembly (see Confirmation of DUF1220 Copy Estimates section) indicates that the CHM1 library is representative of the general population, it cannot be ruled out that some of the changes may be true variants within the human population.

**Table 1:** **Copy number differences in 1q21 between the GRCh37 build and the CHM1 assembly**

Gene name	GRCh37/hg19	CHM1 assembly
*FAM72D*	1	2
*FCGR1A*	1	2
*HYDIN*	0	1
*NBPF16*	1	0
*NBPF24*	1	0
*NBPF25*	1	0
*NBPF26*	0	1
*NOTCH2NL*	1	3
*PDE4DIP*	1	3
*PDZK1*	3	2
*SEC22B*	1	3
*SRGAP2*	1	2
Total 1q21 DUF1220	242	238
DUF1220 CON1	22	17
DUF1220 CON2	13	11
DUF1220 CON3	12	11
DUF1220 HLS1	60	62
DUF1220 HLS2	68	69
DUF1220 HLS3	64	66
DUF1220 Triplets	51	59

Table 1 describes changes in copy number between the GRCh37 and new CHM1 assemblies. The majority of non-DUF1220 changes were gain in copy, with 8 genes previously under represented in GRCh37. Three *NBPF* genes are no longer present, although DUF1220 numbers remained close to the same with DUF1220 copies being mostly redistributed among the remaining *NBPF* genes. This may indicate that the gene loss is an artifact of misassembly rather than true gene copy number differences. Numbers do not include the additional DUF1220 domains and *NBPF* genes added to the 1p11.2 region as a result of the human-specific 1q21.2 segmental duplication described in the text.

**Figure 2:**
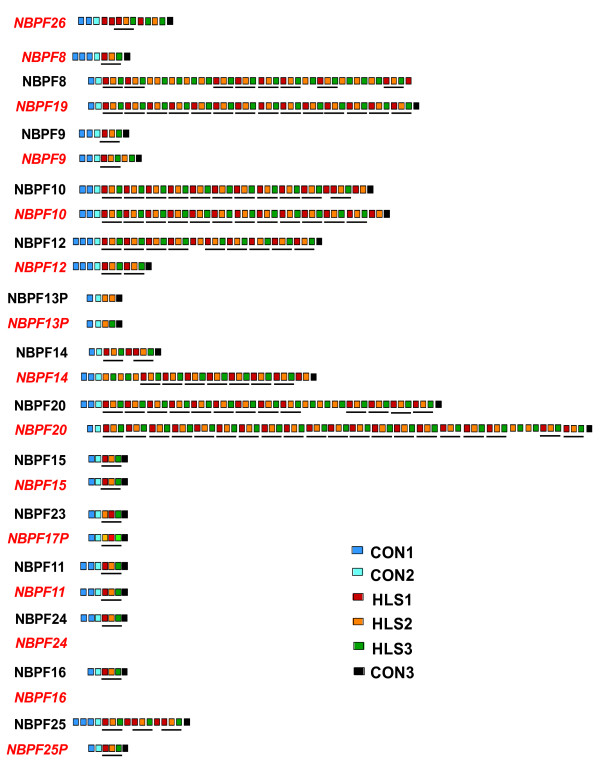
**Organization of the DUF1220 domain and** ***NBPF*** **gene families in the 1q21.1-21.2 region in the GRCh37/hg19 assembly (black) and new CHM1 assembly (red).** Three *NBPF* genes have been lost in the CHM1 assembly, and were likely artifacts of misassembly rather than true differences between the two. Six *NBPF* genes show different DUF1220 copy numbers between builds. The 6 different DUF1220 clades are denoted by colored boxes and DUF1220 triplets are underlined.

The DUF1220-containing *NBPF* family of genes experienced by far the largest number of changes resulting from the CHM1 assembly (Figure 2, Table 2). While there was no significant change in DUF1220 number within the 1q21 region, the domains were redistributed among 11 predicted *NBPF* genes, rather than 13, resulting in sequence changes to 8 *NBPF* genes and the redesignation of many DUF1220 domains to different domain subtypes (hereafter referred to as clades). Finally, two *NBPF* genes were assigned to 1p11.2, resulting in the addition of 21 DUF1220 domains to the haploid genome. This gives a total haploid DUF1220 copy number of 289 domains in the GRCh38 genome build. It is important to note that this is likely the minimum number of copies in the human genome due to the tandemly duplicated nature of these domains and the resulting challenges to sequencing. Current sequencing technology often collapses tandemly duplicated reads into single copies or, less frequently, overestimates the number of copies within these regions. Therefore, the true number of copies in these duplications may be slightly different than that determined by sequencing.

**Table 2:** **Description of NBPF genes in GRCh38 assembly**

Name	Location	No. of DUF1220	No. of DUF1220 triplets
*NBPF1*	1p36.13	7	0
*NBPF2P*	1p36.12	3	0
*NBPF3*	1p36.12	5	0
*NBPF4*	1p13.3	4	0
*NBPF5P*	1p13.3	2	0
*NBPF6*	1p13.3	4	0
*NBPF7*	1p12	2	0
*NBPF8*	1p11.2	8	1
*NBPF26*	1p11.2	13	1
*NBPF23P*	1q21.1	0	0
*NBPF17P*	1q21.1	6	0
*NBPF15*	1q21.1	6	1
*NBPF20*	1q21.1	67	20
*NBPF25P*	1q21.1	6	1
*NBPF10*	1q21.1	42	12
*NBPF12*	1q21.1	11	2
*NBPF13P*	1q21.1	5	0
*NBPF11*	1q21.2	7	0
*NBPF14*	1q21.2	32	7
*NBPF9*	1q21.2	9	1
*NBPF19*	1q21.2	45	14
*NBPF18P*	1q21.3	0	0
*NBPF21P*	3p22.2	1	0
*NBPF22P*	5q14.3	2	0

Table 2 displays a summary of all annotated NBPF regions in the GRCh38 build. There are 23 NBPF-like regions, with 14 NBPF genes and 9 pseudogenes.

**Figure 3:**
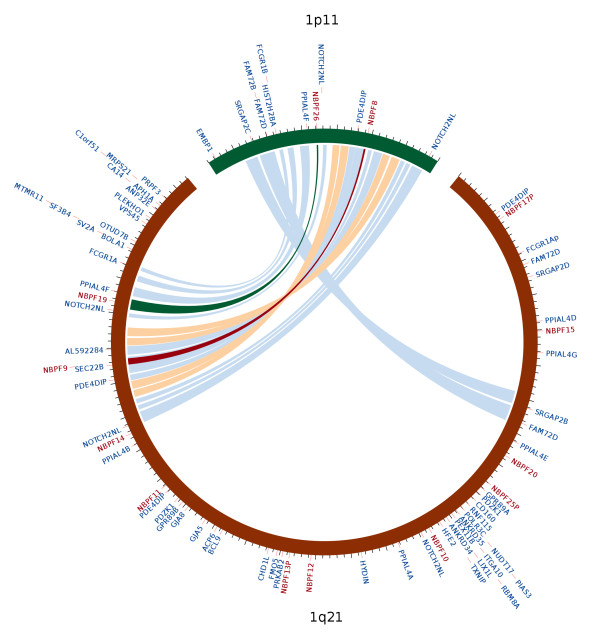
**Comparison of 1q21 to 1p11.2 showing two separate duplication events between the two regions: 1) a segmental duplication between 1q21.2 and 1p11.2 containing 11 genes, including 2 NBPF genes and 2) a smaller duplication from 1q21.1 to 1p11.2**[21].

The two *NBPF* genes newly placed at 1p11.2 are the result of a human lineage specific (HLS) segmental duplication (SD) from the 1q21.2 region to the 1p11.2 region, discovered through sequencing efforts for the CHM1 1q21 region (Figure 3). This SD is not present in other primates and is particularly interesting as both it and its paralog in the 1q21.2 region were identified in Szamalek et al. [19] as the breakpoint regions for a HLS pericentric inversion event. This event is hypothesized to be the catalyst for the expansion of the 1q12 C-band and the hyper-amplification of the HLS DUF1220 triplet in the 1q21 region [20]. The discovery of this SD is an important finding as it helps narrow the time frame in human evolution when the HLS chromosome 1 pericentric inversion took place.

### Confirmation of DUF1220 Copy Number Estimates

#### Digital droplet PCR

DUF1220 clade (subtype) copy number estimates were compared within each *NBPF* gene between the new and old assemblies (Figure 2). DUF1220 domains are subdivided into six clades based on sequence similarity, referred to as conserved (CON) clades 1 through 3 and human lineage specific (HLS) clades 1 through 3. CON1 and HLS1 were analyzed for DUF1220 copy number validation. The CON1 copy number determined by ddPCR of CHM1 DNA was comparable to that seen across multiple control samples, suggesting that the assembly of this clade within *NBPF* genes accurately reflects the general population. The HLS1 copy number, meanwhile, was slightly lower than that seen in the majority of control samples, suggesting that the CHM1 genome may have fewer HLS copies than would be found in healthy individuals. It should be noted that HLS1 DUF1220 domains are highly polymorphic within the population and as such these results do not necessarily suggest that either version of the 1q21 assembly will be more useful for future analysis of copy number-phenotype correlations for this locus. Copy number measurements of *PDE4DIP* as measured by ddPCR reflected those predicted by the molar assembly and mirrored those seen in healthy controls. In addition, a primer set mapping uniquely to *NBPF11* and *NBPF24* (*NBPF* genes differing by only 3 nucleotides) from the GRCh37 assembly was used to determine if the loss of one of these regions was unique to the CHM1 cell line or representative of healthy individuals as well. ddPCR results demonstrate a single copy, confirming loss of *NBPF24* and retention of the one copy of *NBPF11*. Overall, results indicate that the CHM1 assembly generally is representative of healthy individuals and can be used in place of the current human genome build.

#### Irys

**Figure 4:**
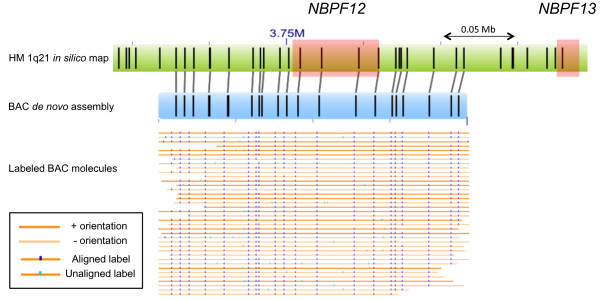
**Single-molecule genome maps (orange) from three hydatidiform mole BACs were assembled** ***de novo*** **into consensus genome maps (blue).** One of the assembled consensus genome maps is shown here (BAC CH17-112A12, blue) and is aligned to an *in silico* map based on the 1q21 sequence assembly described in this paper (green). Locations of the *NBPF12* and *NBPF13* genes on the 1q21 sequence assembly are marked in red. Segment lengths between labels in the *NBPF12* gene are consistent across *in silico* and *de novo* maps.

Single DNA molecules (>150 kb) fluorescently labeled at BspQI sites were used to examine genome segment length within *NBPF* genes in BACs used to produce the new assembly*.* Segment lengths between BspQI labels observed in consensus maps *de novo* assembled from BAC molecules harboring *NBPF10*, *NBPF12* and *NBPF15* (*NBPF12* shown in Figure 4) were consistent to those observed in *in silico* maps of the CHM1 assembly.

### Evolutionary Analysis

**Figure 5:**
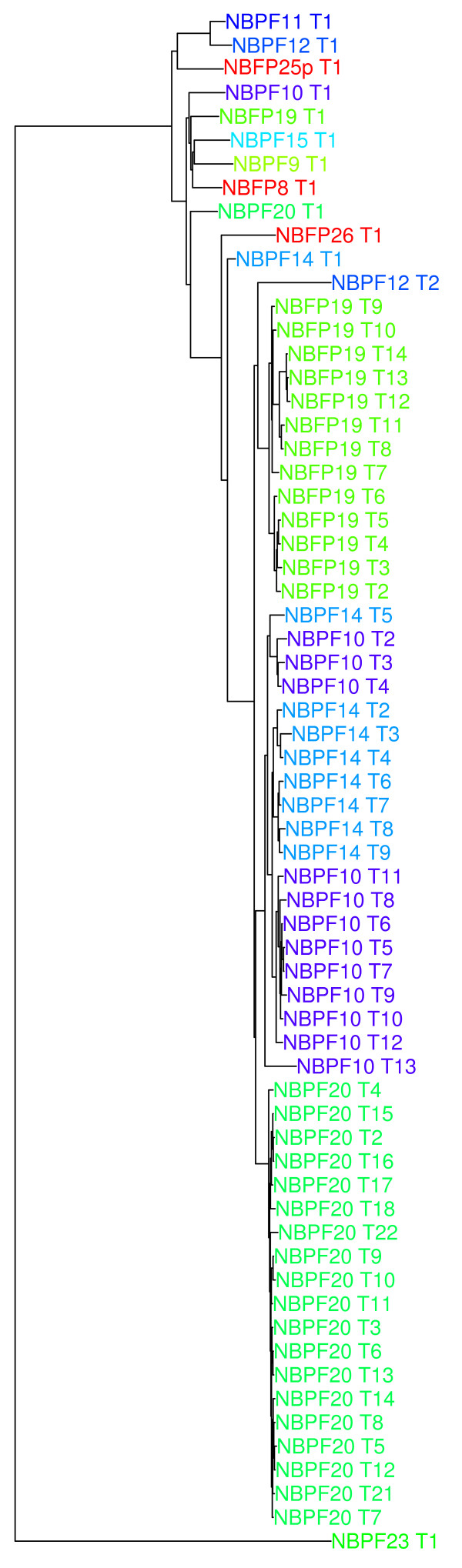
**Phylogeny of DUF1220 triplets in the CHM1 assembly.**

All complete HLS DUF1220 triplet sequences were used to create a phylogenetic tree (Figure 5). The phylogeny reveals first and foremost a confirmation that only *NBPF* genes within the 1q21 region have undergone triplet hyper-amplification. While two new *NBPF*s found to reside in 1p11.2 each contain a HLS DUF1220 triplet, they remain in the unexpanded form and cluster with the first triplets of other *NBPF* genes within the phylogeny. Second, as the 1p11.2 *NBPF* genes are unexpanded when compared to their 1q21.2 counterparts, the most parsimonious conclusion is that the SD from 1q21.2 to 1p11.2 occurred prior to the genomic changes that led to the HLS DUF1220 triplet hyper-amplification. This, combined with the previous discussion of the SD being found to be the breakpoints for the HLS pericentric inversion event, lends more support for the hypothesis put forth recently [20] that the 1q21.1-q21.2 region within the pericentric inversion is a unique genomic environment that allowed the HLS DUF1220 triplet to hyper-amplify.

### Mapping disease samples

**Figure 6:**
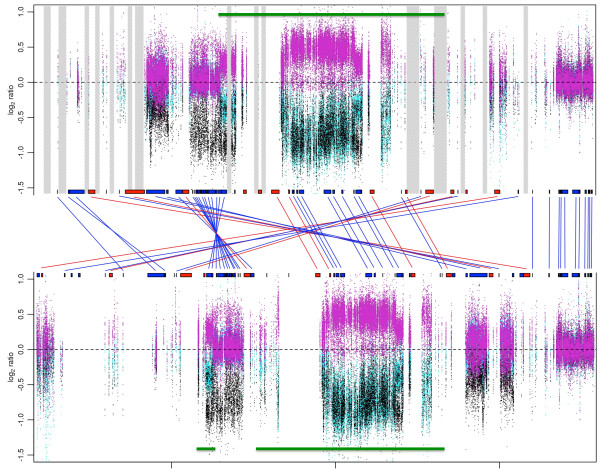
**Comparison of arrayCGH profiles of patients with 1q21 deletions and duplications between the GRCh37/hg19 assembly and the CHM1 assembly.** Samples with known duplications are represented in pink, Type I deletions in blue, and Type II deletions, which are larger than Type I deletions and include the thrombocytopenia-absent radius (TAR) region, in black. Gray vertical regions in the GRCh37/hg19 assembly represent gaps that were eliminated in the CHM1 assembly. Green bars above the GRCh37map and below the CHM1 map indicate the approximate location of the Type I deletion in each assembly. Note that the inverted gene segment in the CHM1 assembly requires a two-deletion event rather than single-deletion event to explain the Type I deletion mapping pattern. Tick marks at the bottom of the figure are separated by 2 Mb; the GRCh37 assembly starts at 142,000,000 and the CHM1 assembly starts at 0.

ArrayCGH results of patient samples from the Baylor College of Medicine with known 1q21 CNVs [17] were mapped to the 2009 assembly and the new WUSTL CHM1 assembly. Figure 6 shows a comparison of these data plotted on the 2009 assembly (upper image) and the CHM1 assembly (lower image). Regions affected by these copy changes overlapped, but were not identical, between assemblies. In addition, there are genes that do not appear to be affected by deletion/duplication in the 2009 assembly (or are not present in that assembly) that fall in affected regions in the CHM1 1q21 assembly (not shown).Of note, one segment of the 2009 assembly that contains multiple genes is inverted in the new assembly (Figure 1). By mapping patient data to this new assembly, it was discovered that this inversion, while representative of the CHM1 genome, may be polymorphic in the human population (or may be inverted uniquely in the CHM1 genome). This conclusion is based on the fact that in the new CHM1 assembly, samples with Type I deletions (a class of deletions found in the distal 1q21 region) have a contiguous gene deletion region, and an additional region deleted proximally that is not contiguous, suggesting two deletion events (Figure 6). In the 2009 assembly, these two deleted regions are contiguous, suggesting one deletion event rather than two. As a single-deletion event is more likely than multiple deletions events, it is likely that this inversion occurred in the CHM1 genome but may not be the more common allelic form in the human population.

By remapping array results for 40 patients with 1q21 CNVs to the new assembly, regions affected by deletions and duplications were identified with better certainty and precision. In the future, the more accurate and complete 1q21 assembly will also allow for better mapping of breakpoints of these copy number changes, which will in turn aid in localizing disease-causing genes and regulatory loci, and in the development of a morbidity map of the region.

## Conclusions

This investigation demonstrated that resources developed from a haploid hydatidiform mole genome could effectively be used to complete assembly of chromosome 1q21, one of the most complex and evolutionarily dynamic regions of the human genome. Completing this region also has significant implications for studying human disease given the numerous disorders associated with CNVs, mutations and chromosomal aberrations in the 1q21 region. Additionally, the complete 1q21 assembly will play an integral role in studies of human evolution, as 1q21 contains the majority of the 289 DUF1220 protein domain copies, 160 of which were added specifically to the human genome since the Homo/Pan divergence [15][20]. The new 1q21 assembly has already led to the discovery of a novel copy of *SRGAP2*, a gene in the 1q21 region that may be important for the elaboration of neuronal processes in the human brain [21]. Efforts to localize disease-causing genes and regulatory regions that have previously been hindered by the incomplete nature of the 1q21 assembly and inaccuracies in the region may now move forward with a complete and reliable map to identify causative sequence variations.

## Methods

### Hydatidiform mole

Hydatidiform moles are human conception abnormalities that most often arise from the fertilization of an anucleate ovum by a single X-bearing sperm. Subsequent diploidization results in a 46 XX karyotype in which all allelic variation has been eliminated allowing the unambiguous delineation of duplicated DNA as well as haplotype characterization. The hydatidiform mole (CHM1) BAC library (CHORI-17) was previously created by the Children’s Hospital Oakland Research Institute BACPAC Resource by Mikhail Nefedov in Pieter de Jong’s laboratory. The library was prepared from a well-characterized haploid cell line (CHM1htert) from Dr. Urvashi Surti, Director of the Pittsburgh Cytogenetics, laboratory, using the cloning approach described in Osoegawa et al. [22]. This library was used for subsequent analysis.

### Sequence finishing and assembly

A minimum tiling path of single haplotype clones was selected based off of a fingerprint map and alignment of existing BAC end sequences (BES) to span the 1q21.1-q21.2 region of interest. Sequences were generated to cover each BAC insert as described below. The clones were pooled prior to sequencing in groups of 25, in equal molar ratio, and a single 454 fragment library and a single 3730 plasmid library were produced. This approach leveraged the high throughput, unbiased 454 data with the 3730 data, which provides long-range linkage, long reads for assembly, and template availability. The 454 pools were sequenced to greater than 25× coverage, and 3730 libraries to a coverage of 4×. In addition, BACs difficult to resolve due to multiple paralog content had individual 3730 libraries created and sequenced.

The data was assembled using a *de novo* assembly approach using both pcap [23] and newbler (Roche 454 software package) assembly algorithms to assemble the data. These assemblies were then compared to one another as well as to the human reference sequence, to further guide the assembly and resolve any sequencing ambiguities. This approach has been applied extensively to whole genome bacterial projects in the size range of 5 Mb, with great success, as well as clone pools in human structural variation fosmid projects [21]. An automated improvement process called prefinishing was performed to choose directed work for low quality regions and gaps, and then a manual process of finishing the regions to a level of less than 1 error per 10,000 bp was employed. At the end of this process, a high quality product suitable for identification of sequence differences between the reference sequence and the single haplotype was achieved. The new assembly can be found at accession number JH636052.4.

### *NBPF* gene annotation

All DUF1220/*NBPF* homologous regions were evaluated using the criteria published in O’Bleness et al. [20]. Through a collaboration between the Sikela laboratory, RefSeq at NCBI, and the HUGO Gene Nomenclature Committee, a consensus gene nomenclature was decided upon. This nomenclature is used in the GRCh38 release of the human genome.

### Analyses of segmental duplications and DUF1220 HLS triplet expansion events

Evaluation of the 1q21 to 1p11 duplication events was generated by aligning the CHM1 1q21 region to the CHM1 1p11 region using the Exonerate alignment tool with the genome to genome option [24] and visualized in GBrowse for manual annotation and confirmation. The largest and highest scoring alignments were plotted using the Circos visualization tool (Figure 3) [25]. Evaluation of the relationship between the DUF1220 HLS triplet (hls1-hls2-hls3) sequences in each NBPF gene was performed by aligning each DUF1220 HLS triplet using the PRANK multiple sequence aligner [26] and generating a phylogenetic tree using the APE package in R (Figure 5) [27].

### Mapping of disease samples to the new 1q21 assembly

40 patient samples from the Baylor College of Medicine with 1q21 microduplications or microdeletions were identified by array comparative genomic hybridization (arrayCGH) using probes specific to the 2009 1q21 assembly. Arrays were previously constructed using Agilent custom array capabilities, designed and processed as described in Dumas et al. [17]. Probes from the initial run based on the old assembly were re-mapped to the new assembly in order to identify loci affected by deletion/duplication status that vary by assembly map.

### Droplet digital PCR confirmation of copy number estimates in the new 1q21 assembly

Droplet digital PCR (ddPCR), a third-generation PCR technique [28], was used to check the copy number estimates of multiple loci in the new assembly. DNA was extracted from CHM1htert cell pellets, provided by Dr. Urvashi Surti, using a Qiagen DNA extraction kit following manufacturer’s protocols. Extracted DNA was digested with the restriction enzyme DDE1. Digested DNA was then added to a PCR mix according to the manufacturer’s protocol including fluorescently tagged probes specific to the region of interest (separate reactions for conserved clade 1 (CON1) (Left ‘AATGTGCCATCACTTGTTCAAATAG’ , Right – ‘GACTTTGTCTTCCTCAAATGTGATTTT’ , Hyb – ‘CATGGCCCTTATGACTCCAACCAGCC’), human lineage specific clade 1 (HLS1) (Left – ‘GCTGTTCAAGACAACTGGAAGGA’, Right - ‘GGGAGCTGCTGGAGGTAGT’ , Hyb – ‘AGAGCCTGAAGTCTTGCAGGACTCAC’), PDE4DIP (Left – ‘GCCTTATTAGCATCCCAAGACAA’ , Right – ‘CCCTGAACAGCCTTTCCTTCT’ , Hyb – ‘CATGCTGTGAAGAAGTCGGTCTACCCCAC’), and a unique region mapping to *NBPF11* (Left - ‘GGAAAGTCGGGTTTGTGAGA’, Right – ‘TGGCACAACATCCTGGAATA’ , Hyb – ‘ACAACAGAGGAGAGCGGAGA’) and to a reference sequence of known copy number, RPP30 (Left – ‘GATTTGGACCTGCGAGCG’, Right – ‘GCGGCTGTCTCCACAAGT’ , Hyb – ‘TTCTGACCTGAAGGCTCTGCGC’). Oil droplets containing this mixture were produced using a BioRad droplet generator, resulting in over 14,000 droplets per well. Droplets were then subject to a thermocycling protocol with an annealing temperature of 56°C and read single-file on a droplet reader to compare fluorescence of the target and reference in each droplet. Results were merged to produce a final copy number estimate and this estimate was compared to that provided by the new assembly and to ddPCR results examining the same loci in healthy controls from the Coriell dataset.

### Confirmation of *NBPF* data using Irys technology

The Irys platform automates high-resolution genome mapping by imaging labeled single DNA molecules in nanochannels. Three hydatidiform mole BACs (CH17-112A12, CH17-353B19 and CH17-382H24) containing *NBPF12*, *NBPF10* and *NBPF15*, respectively, were selected for genome mapping to validate the sequence assembly of the chr1q21 region. BAC DNA was extracted with Large Construct kit (Qiagen, Valencia, CA) and 300 ng of purified BAC DNA were used for nicking and labeling according to the irysPrep protocol (BioNano Genomics, San Diego, CA). In brief, 300 ng of purified BAC DNA were incubated in a 10 μL nicking reaction at 37°C with 7 U Nt.BspQI (NEB, Ipswich, MA) for two hours followed by heat inactivation of the nicking enzyme at 80°C for 20 minutes. Five microliters of labeling master mix, consisting of 1.5× labeling buffer, 1.5× labeling mix (BioNano Genomics) and 1 U Taq polymerase (NEB), was added to the heat-inactivated nicking reaction mixture and this labeling reaction mixture was incubated at 72°C for an hour. The nicked and labeled DNA was repaired by PreCR® repair mix that contained 10 mM dNTP mix (NEB). One microliter of stop solution (BioNano Genomics) was added to stop the repair reaction. Lastly, the nicked, labeled and repaired DNA was stained overnight with DNA stain (BioNano Genomics). The nicked, labeled, repaired and stained BAC DNA samples (20 μL each) were combined before they were loaded on the IrysChip for genome mapping on the Irys system (BioNano Genomics).

Image detection, genome map alignment, and assembly were performed using software tools developed in-house and packaged into IrysView at BioNano Genomics. Briefly, the DNA backbone and fluorescent labels were detected, integrated, and converted into single-molecule genome maps. *De novo* assembly of genome maps was performed using a graph-based assembler. Consensus genome maps were then aligned to an *in silico* map based on the 1q21 sequence assembly.

## Abbreviations

ArrayCGH: Array comparative genomic hybridization
DUF1220: Domain of unknown function 1220
NBPF: Neuroblastoma breakpoint family
Hg19: Human genome version 19
GRCh37: Genome Reference Consortium Human Build 37
BAC: Bacterial artificial chromosome
HM: Hydatidiform mole
HLS: Human lineage-specific
SD: Segmental duplication
ddPCR: Droplet digital PCR
CON1: Conserved clade 1
PDE4DIP: Phosphodiesterase 4D-interacting protein
HLS1/2/3: Human lineage specific clades 1/2/3
CHORI-17: Children’s Hospital Oakland Research Institute-17 (name of Hydatidiform Mole BAC Library)
BES: BAC end sequences
WUSTL: Washington University St. Louis.
